# Bayesian joint models with INLA exploring marine mobile predator–prey and competitor species habitat overlap

**DOI:** 10.1002/ece3.3081

**Published:** 2017-06-07

**Authors:** Dinara Sadykova, Beth E. Scott, Michela De Dominicis, Sarah L. Wakelin, Alexander Sadykov, Judith Wolf

**Affiliations:** ^1^ Institute of Biological and Environmental Sciences University of Aberdeen Aberdeen UK; ^2^ School of Biological Sciences Queen's University Belfast Belfast UK; ^3^ National Oceanography Centre Liverpool UK; ^4^ The Centre for Ecological and Evolutionary Synthesis University of Oslo Oslo Norway

**Keywords:** Besag models, bio‐physical habitats, hurdle models, integrated nested Laplace approximation, mobile marine species, spatial joint modeling, spatial niche selection, stochastic partial differential equations, zero‐inflated models

## Abstract

Understanding spatial physical habitat selection driven by competition and/or predator–prey interactions of mobile marine species is a fundamental goal of spatial ecology. However, spatial counts or density data for highly mobile animals often (1) include excess zeros, (2) have spatial correlation, and (3) have highly nonlinear relationships with physical habitat variables, which results in the need for complex joint spatial models. In this paper, we test the use of Bayesian hierarchical hurdle and zero‐inflated joint models with integrated nested Laplace approximation (INLA), to fit complex joint models to spatial patterns of eight mobile marine species (grey seal, harbor seal, harbor porpoise, common guillemot, black‐legged kittiwake, northern gannet, herring, and sandeels). For each joint model, we specified nonlinear smoothed effect of physical habitat covariates and selected either competing species or predator–prey interactions. Out of a range of six ecologically important physical and biologic variables that are predicted to change with climate change and large‐scale energy extraction, we identified the most important habitat variables for each species and present the relationships between these bio/physical variables and species distributions. In particular, we found that net primary production played a significant role in determining habitat preferences of all the selected mobile marine species. We have shown that the INLA method is well‐suited for modeling spatially correlated data with excessive zeros and is an efficient approach to fit complex joint spatial models with nonlinear effects of covariates. Our approach has demonstrated its ability to define joint habitat selection for both competing and prey–predator species that can be relevant to numerous issues in the management and conservation of mobile marine species.

## 1 INTRODUCTION

The marine environment is changing rapidly due to climate change (Burrows et al., 2011) and increasing anthropogenic activities (Wakelin, Artioli, Butenschön, Allen, & Holt, 2015) including large‐scale energy extraction (tidal, wave, and wind; Shields & Payne, 2014). Understanding how usage of spatial habitat of highly mobile marine species may change with these pressures is essential for sustainable management of their populations. Mobile species can be in competition with one another and are involved in predator–prey relationships. As bio/physical conditions change, it is essential to predict the effect of habitat changes. The habitat changes can occur on both individual species’ spatial distribution level and the combination of species spatial habitat overlap. We suggest that using joint models, as compared to single‐species models, will allow a more complete understanding of the nature of multiple species habitat selection and bio/physical covariate effects. Joint models combine information across species and reduce variability by assuming a shared spatial structure (referred in this paper as “common spatial trends”) informed by more data (Illian et al., 2013; Reynolds et al., 2009). Identifying common spatial trends is vital for quantifying the degree of spatial overlap for competing or predator–prey species and may provide a basis for understanding common spatial habitats.

Modeling large and complex spatial datasets is also computationally challenging due to the inclusion of a spatial correlation structure (Illian et al., 2013). Considering joint models and nonlinear relationships between species distributions and habitat variables leads to even higher computational cost. Moreover, species spatial data add to model complexity because they are often characterized by excess of zeros. These zeros can occur for multiple reasons: false negatives (not seen when present, being difficult to observe) or because species are rare or highly aggregated in both space and time.

To deal with the above issues and investigate whether there are common spatial trends between competing and predator–prey species, we considered a Bayesian hierarchical joint modeling approach with integrated nested Laplace approximation (INLA) that substantially reduce the computational cost of fitting complex spatial models (Rue, Martino, & Chopin, 2009) and applied the methodology to the single‐species and joint‐species spatial and spatiotemporal hurdle and zero‐inflated models. We fit these models to the spatial patterns of eight mobile marine competing and predator–prey species: grey and harbor seals, harbor porpoise, common guillemot, black‐legged kittiwake, northern gannet, Atlantic herring and sandeels. The modeling approach used six physical and biologic variables that are predicted to change with climate change (Holt, Butenschon, Wakelin, Artioli, & Allen, 2012; Holt, Hughes et al., 2012) and energy extraction (De Dominicis, O'Hara Murray, & Wolf, 2017; Van der Molen, Ruardij, & Greenwood, 2016) and have been considered to be important habitat variables for a range of species: temperature, levels of maximum and cumulative primary production, levels of stratification and aspects of speed, and both horizontal and vertical (Bailey & Thompson, 2010; Bost et al., 2009; Carroll et al., 2015; Schick et al., 2011; Scott et al., 2010; Sharples, Scott, & Inall, 2013). This study sets out to identify which of the bio/physical variables play the most significant role in determining habitat preferences of the selected marine species, defines habitat preferences, measures estimated effect of the bio/physical variables on the selected species, and investigates whether there are common spatial trends for competing and predator–prey species.

## 2 DATA DESCRIPTION

### 2.1 Study area

The study area was defined as covering the North Sea and the UK continental shelf as the area between 48° and 62° North and 10° West and 12° East.

### 2.2 Study species

#### 2.2.1 Grey and harbor seal usage density maps

The seal usage density maps (Fig. S1 in Appendix S1, top panel) represent estimated at‐sea distributions of grey seals (*Halichoerus grypus*) and harbor seals (*Phoca vitulina*) (×10^2^) in each 5 × 5 km grid square around the UK. These maps synthesize over 20 years of telemetry and survey count data. Usage is not seasonal but represents habitats used over the entire year. More details can be found in Jones, McConnell, Sparling, and Matthiopoulos (2013).

#### 2.2.2 Harbor porpoise density maps

The predicted density maps for harbor porpoise (*Phocoena phocoena*) in 1994 and 2005 show porpoise density in individuals per km^2^ (Fig. S1 in Appendix S1, bottom panel). These maps are based on the data from the cetacean surveys which were performed during July of each year known as SCANS (Hammond et al., 2013).

#### 2.2.3 Atlantic herring abundance

Maps of the herring (*Clupea harengus*) abundance (Fig. S2 in Appendix S1) represent the herring mean abundance (in 100 million individuals) for ages 1, 2, and 3 in each 56 × *56 km grid cell for the combination of the years 2003–2009 and 2013–2014. The dataset includes survey effort that is given as grid cell coverage by cruise tracks (in nautical miles) and are based on annual herring acoustic surveys preformed in July of each year (ICES 2015, 2016).

#### 2.2.4 Seabird observational at‐sea survey data

The European seabird at sea database (ESAS) presents ship‐based survey observations of common guillemot (*Uria aalge*), black‐legged kittiwake (*Rissa tridactyla*), and northern gannet (*Morus bassanus*) (Fig. S3 in Appendix S1, right panel) made over a period of more than 30 years (1979–2011) (Kober et al., 2010, 2012). The dataset includes a trip, position, and full dates. For this analysis, we extracted only those birds sitting on the water as those were assumed to have been recently foraging. We created two seasonal outputs of observational data representing spring (March, April, May, and June) and summer (July, August, September, and October) months covering breeding and non‐breeding behavioral periods. Due to the ESAS dataset having some areas with much more frequent survey effort than others, we constructed a survey effort variable that determines how many times each grid cell was visited (with the grid size of 300 × 300 m as transects were spaced approximately at 300 m intervals).

#### 2.2.5 Seabird density maps

Due to the high number of zeros in the observational data, we also used seabird density maps (×10^2^) (Fig. S3 in Appendix S1, left panel) which were based on the bird observation data using Poisson kriging and represent predicted density of common guillemot, black‐legged kittiwake, and northern gannet in each 6 × 6 km grid cells across the 28‐year data (1979–2006). The seabird density maps, which use flying seabirds as well as seabirds sitting on the water, were made taking into account unequal sampling effort in space and time. More information can be found in Kober et al. (2010).

#### 2.2.6 Sandeel observation data

Sandeel data from the CPR surveys (Edwards et al., 2011) show observations of sandeel larval abundance (number/m^3^) (*Ammodytidae sps*) (Fig. S4 in Appendix S1, right panel) made over a period of 58 years (1948–2005). Larval distributions were used to represent the range of habitat areas that both adult and juvenile sandeels can inhabit. In this paper, we used years 1989–2005 to cover a representative average climate period of the comparative bio/physical data. A trip, position, and full dates (time, day, month, and year) were included in the dataset (find the detailed description of the data in Edwards et al. (2011)).

In a similar way to the seabird data, we constructed an effort variable that determines how many times each grid cell was visited (with the grid size of 300 × 300 m).

#### 2.2.7 Sandeel density maps

Again due to such high number of zeros in the observational data, we created sandeel density maps (×10^2^) (Fig. S4 in Appendix S1, left panel) in 7 × 7 km grid mesh across the 16‐year data (1989–2005) using Poisson kriging, which takes into account unequal survey efforts and is suitable to the observation data that are heterogeneously distributed (Kober et al., 2010). Poisson kriging was applied separately to the two seasons: spring and summer.

### 2.3 Physical environmental variables

Data on six biologic and physical environmental variables have been provided from runs of the NEMO‐ERSEM 3D‐coupled hydrodynamic‐ecosystem model (Edwards, Barciela, & Butenschön, 2012; O'Dea et al., 2012; Wakelin, Artioli, Butenschön, & Holt, 2017). These are a subset of variables that are expected to change with both climate change (Holt, Butenschon et al., 2012; Holt, Hughes et al., 2012; Wakelin et al., 2015) and potentially as a consequence of large‐scale energy extraction for renewable energy (De Dominicis et al., 2017; Van der Molen et al., 2016) and also that capture key changes in habitats (Figs. S5, S6 in Appendix S1): bottom temperature (BT) (°C), maximum chlorophyll_*a* (CHL) (mgC/m^3^), net primary production (NPP) (mgC/m^2^/day), potential energy anomaly (PEA) (J/m^3^) (which is the energy required to mix the water column completely), depth‐averaged horizontal current speed (SP) (m/s), and depth‐averaged vertical velocity (DVV) (m/day).

All the variables were given on a regular 7 × 7 km^2^ grid for two seasons. The first season (“spring season”) represents spring and early summer (breeding/juvenile development periods for many species) and includes March, April, May, and June. The second season (“summer season”) representing post‐breeding and includes July, August, September, and October. All the data were given as climatological means across 25 years (1989–2014).

### 2.4 Data manipulations

#### 2.4.1 Data with excess zeros

Table S1 (Appendix S1) shows the percentage of zeros in the observational and final datasets. Due to the high occurrence of zeros in the observed data, we removed the trips that had only zero observations (Table S1 in Appendix S1).

#### 2.4.2 Grid resolution

The usage maps (grey seals and harbor seals) and density maps (harbor porpoise, northern gannet, common guillemot, and black‐legged kittiwake) were transferred to a regular 7*7 km^2^ grid using bilinear interpolation for computational optimization purposes. Grids with a finer resolution (6*6 km, 5*5 km, and 1*1 km) have been checked for representative species (black‐legged kittiwake, grey seals, and harbor porpoise, respectively) to assess whether the results are influenced by the fineness of the grid, but all spatial scales produced nearly the same results in terms of the habitat preferences, model selection, and estimated common spatial trends. Therefore, only 7*7 km^2^ grid results are presented.

#### 2.4.3 Combining species and bio/physical datasets

The point locations of the density and usage maps based on the 7*7 km^2^ grid matched the locations of the bio/physical variables based on the regular 7*7 km^2^ grid. For the observation data and the abundance/density/usage maps with finer resolution grids, we used predictive joint modeling with misalignment (Krainski, Lindgren, Simpson, & Rue, 2015) to predict bio/physical variables on the species locations (see the Joint Modeling with Misalignment section).

## 3 METHODS

### 3.1 Integrated nested Laplace approximation

Integrated nested Laplace approximation (INLA) is a computationally efficient method for fitting complex spatial models, which was created as an alternative to Markov Chain Monte Carlo (MCMC) methods (Lindgren & Rue, 2015; Rue et al., 2009, 2014). INLA may be used to fit a large class of latent Gaussian models in a Bayesian framework. A spatial effect is included in INLA models to account for spatial autocorrelations. The INLA approach is faster than MCMC and at the same time flexible and accurate (Rue et al., 2009). Here, we employ INLA to fit single and joint, zero‐inflated and hurdle, spatial and spatiotemporal models.

### 3.2 Models

Both hurdle (Cragg, 1971) and zero‐inflated models (Lambert, 1992) have been developed to manage high occurrence of zeros in the observed data. Both models can be considered as mixture models; however, these models have an important difference in how zero observations are interpreted. A zero‐inflated model assumes that the zero observations have two different origins: structural and sampling (Hu, Pavlicova, & Nunes, 2011). Sampling zeros are due to the usual Poisson (or negative binomial) distribution, which assumes that these zeros happened by chance, whereas structural zeros are due to some specific structure in the data, for example, due to the habitat being unsuitable and the species not being present. A hurdle model considers all zeros are generated by one process with only structural zeros. Both model types are widely used to model count data, whereas the hurdle continuous models (Krainski et al., 2015) are especially useful to model density data.

#### 3.2.1 Zero‐inflated spatiotemporal model

A zero‐inflated model (Lambert, 1992) is a mixture distribution of a Poisson (negative‐binomial) distribution and a point mass at zero. Here, we present a zero‐inflated Poisson model. A full description of the zero‐inflated negative‐binomial model can be found in Greene (1994). For response variable ***y***, let $y_{st,i}^{c}$ denote counts of a species ***i*** at location ***s*** and period of time ***t***, where period can represent either year, month per year, or season per year. We assume that

$$y_{st,i}^{c}∼\left\{\begin{array}{cc} 0 & \text{with probability}p_{st,i}\\ \text{Poisson}\left(\lambda_{st,i}\right) & \text{with probability}1-p_{st,i} \end{array}\right.$$

where λ_*st*,*i*_ is a Poisson mean function, *p*_*st*,*i*_ is a zero‐inflation parameter, for the ***t***th period of time, the ***s***th spatial location and the ***i***th species. The resulting distribution is then expressed as:

$$\begin{array}{cc} P\left(y_{st,i}^{c}=0\right) & =p_{st,i}+\left(1-p_{st,i}\right)e^{-\lambda_{st,i}}\\ P\left(y_{st,i}^{c}=j\right) & =\left(1-p_{st,i}\right)\frac{e^{-\lambda_{st,i}}\lambda_{st,i}^{j}}{j!},j=1,2,\ldots \end{array}$$

where the parameters are modeled by:

(1a) $$\begin{array}{cc} \log\left(\lambda_{st,i}\right) & =\beta_{1,i}+\sum_{k_{1}}f_{\lambda k_{1},i}\left(x_{k_{1}s,i}^{\prime }\right)+f_{\lambda T,i}\left(t_{s,i}\right)+f_{\lambda E,i}\left(\eta_{s,i}\right)+\theta_{\lambda s,i}\left(s_{i}\right)+u_{i}\left(s_{i}\right) \end{array}$$

(1b) $$\begin{array}{cc} \text{logit}\left(p_{st,i}\right) & =\beta_{2,i}+\sum_{k_{2}}f_{pk_{2},i}\left(x_{k_{2}s,i}^{\prime }\right)+f_{pT,i}\left(t_{s,i}\right)+f_{pE,i}\left(\eta_{s,i}\right)+\theta_{ps,i}\left(s_{i}\right)+v_{i}\left(s_{i}\right) \end{array}$$

**Figure 1:**
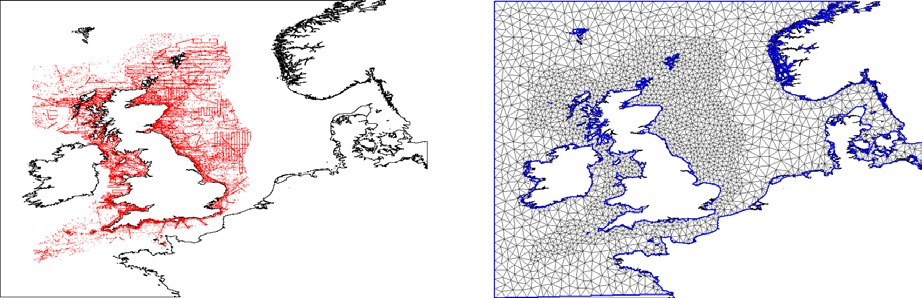
Locations of the black‐legged kittiwake (red dots) (left) and INLA mesh for the single black‐legged kittiwake SPDE model (right)

Here, β_1,*i*_ and β_2,*i*_ are separate means for each species and $x_{k_{1}s,i}^{\prime }$ and $x_{k_{2}s,i}^{\prime }$ are covariates that vary spatially (but not temporally, see the data section). $\sum_{k_{1}}f_{\lambda k_{1},i}\left(x_{k_{1}s,i}^{\prime }\right)$ and $\sum_{k_{2}}f_{pk_{2},i}\left(x_{k_{2}s,i}^{\prime }\right)$ are sums over different combinations of the bio/physical variables’ effects for each species (we considered all possible combinations of the covariates excluding highly correlated (>0.6) variables), where the covariates’ effects are modeled as smooth functions $f_{\lambda k_{1},i}\left(.\right)$ and $f_{pk_{2},i}\left(.\right)$ as first‐order or second‐order random walk processes (RW1 or RW2) to pick up smooth fluctuations (Rue & Held, 2005). RW1 or RW2 was selected using the deviance information criterion (DIC). The period variable (that shows either year, month per year, or season per year) and the effort variable (amount of sampling) are represented by $t_{s,i}$ and η_*s*,*i*_ respectively. We fit smooth functions $f_{\lambda T,i}\left(.\right),f_{pT,i}\left(.\right),f_{\lambda E,i}\left(.\right),$ and $f_{pE,i}\left(.\right)$ as random walk processes of either order 1 or 2 (RW1 or RW2) to them using DIC to select either RW1 or RW2. The random error terms are given by $u_{i}\left(s_{i}\right)$ and $v_{i}\left(s_{i}\right)$. The spatially structured effects that describe the spatial autocorrelation not explained by the covariates are given by $\theta_{\lambda s,i}\left(s_{i}\right)$ and $\theta_{ps,i}\left(s_{i}\right)$ and are modeled by a Gaussian field through the stochastic partial differential equation (SPDE) approach (Lindgren, Rue, & Lindstrom, 2011). SPDE is a computationally effective approach especially useful when dealing with point‐reference data (e.g., continuous data that involve point samples from a continuous spatial distribution, such as birds’ observations in this paper). The key idea of the SPDE approach consist in defining the continuously indexed Matern Gaussian field (GF) (Blangiardo, Cameletti, Baio, & Rue, 2013; Lindgren et al., 2011) as a discreetly indexed spatial random process (GMRF) using piece‐wise linear basis functions defined on a triangulation of the domain of interest. SPDE provides a representation of the whole spatial process that varies continuously in the considered domain (Blangiardo et al., 2013; Lindgren et al., 2011). Figure 1 shows the mesh that was used to approximate the spatial fields. Note that the mesh was extended beyond the study area (where there are no physical boundaries) to avoid a boundary effect where a variance is twice large than within the domain. The SPDE is rather complex approach and its explanation requires a long description; therefore, the reader is referred to the original paper (Lindgren et al., 2011) for more details. We considered the SPDE approach here because the approach is computationally efficient, the data were modeled considering their exact locations (instead of being aggregated into cells), and the approach provided inference about the entire process defined on continuous domain of interest (Lindgren, 2013).

When the model is fitted jointly to few species, the spatial effect between the species is going to be proportional, that is, $\theta_{\lambda s,i_{1}}\left(s_{i_{1}}\right)=\zeta_{\lambda }\theta_{\lambda s,i_{2}}\left(s_{i_{2}}\right)$ and $\theta_{ps,i_{1}}\left(s_{i_{1}}\right)=\zeta_{p}\theta_{ps,i_{2}}\left(s_{i_{2}}\right)$ for any two species *i*_1_ and $i_{2}$, where ζ_λ_ and ζ_*p*_ are parameters to be estimated.

#### 3.2.2 Hurdle spatial and spatiotemporal models

A hurdle model is a two‐component model and these two components of the model are assumed to be functionally independent (Cragg, 1971). The “hurdle” may present any value, but in this study we set the hurdle value at zero. The first part of the hurdle model presents a binary component that generates zeros and ones, where zero corresponds to the zero values and one correspond to positive values. The second part of the model presents an amount component that generates non‐zero values.

For the response variable ***y***, let ***y***_*st*,*i*_ denote either density, usage, abundance, or counts of a species ***i*** at location ***s*** and period of time ***t*** (where period can represent either year, month per year, or season per year). The occurrence variable is defined as $z_{st,i}$ :

$$z_{st,i}=\left\{\begin{array}{cc} 1 & \text{if}y_{st,i}>0\\ 0 & \text{otherwise} \end{array}\right.$$

and the amount variable $y_{st,i}^{A}$ is given by:

$$y_{st,i}^{A}=\left\{\begin{array}{cc} NA & \text{if}\;y_{st,i}=0\\ y_{st,i} & \text{otherwise} \end{array}\right.$$

We then use a logistic regression for the binary processes, a zero‐truncated Poisson (ZTP) model for the positive counts and Gamma model for the positive density, usage, or abundance data:

$$z_{st,i}∼\text{Bernoulli}\left(p_{st,i}\right)$$

$$y_{st,i}^{A}∼\left\{\begin{array}{cc} \text{Gamma}\left(a_{st,i},b_{st,i}\right) & \text{if}y_{st,i}^{A}\text{are density}/\text{usage}/\text{abundance}\\ \text{ZTP}\left(\lambda_{st,i}\right) & \text{if}y_{st,i}^{A}\text{are positive counts} \end{array}\right.$$

We consider that:

(2a) $$E\left(y_{st,i}^{A}\right)=\frac{a_{st,i}}{b_{st,i}}=\mu_{st,i}$$

(2b) $$\text{Var}\left(y_{st,i}^{A}\right)=\frac{a_{st,i}}{{\left(b_{st,i}\right)}^{2}}=\frac{{\left(\mu_{st,i}\right)}^{2}}{\phi }$$

$\text{if}y_{st,i}^{A}∼\text{Gamma}\left(a_{st,i},b_{st,i}\right).$ Here, ϕ is a precision parameter. Then, the linear predictors $\log\left(\lambda_{st,i}\right)$ and $\log\left(\mu_{st,i}\right)$ for the spatiotemporal models are defined by equation (1a) and the linear predictor to the first component $\text{logit}\left(p_{st,i}\right)$ is defined by equation (1b). These linear predictors for the spatial non‐temporal models are defined by the same equations excluding the period effect. The spatial effects $\theta_{\lambda s,i}\left(s_{i}\right),\theta_{\mu s,i}\left(s_{i}\right),$ and $\theta_{ps,i}\left(s_{i}\right)$ are modeled by Gaussian Markov random field through either the SPDE approach (Lindgren et al., 2011) briefly described in the “zero‐inflated spatiotemporal model” section above or Besag–York–Mollie (BYM) specification, where the spatially structured effects are modeled using an intrinsic conditional autoregressive structure (iCAR), a zero‐mean Gaussian Markov random field (GMRF) (Besag, York, & Mollie, 1991; Bivand, Gomez‐Rubio, & Rue, 2015; Rue & Held, 2005). In addition to the spatially structured effect, the BYM model also includes an additional unstructured random term to account for independent region‐specific noise. All the intrinsic Gaussian Markov random fields models were scaled to a unit generalized variance to avoid that the precision parameters of these models have different interpretation (Sørbye & Rue, 2014). For the BYM approach, we used regular 7*7 km^2^ mesh and two locations were considered to be neighbors if they were closer than distance *R* apart. The *R* values were selected so that either four or eight nearest neighbors were included in consideration. The results were nearly identical and therefore we show only eight‐neighbors results (see section 4 below). For more details about the Besag–York–Mollie (BYM) specification, see Besag et al. (1991), Rue and Held (2005), and Bivand et al. (2015).

Although the BYM approach is common for the areal datasets (e.g., aggregated quantiles for each areal unit, such as densities, usage, or abundance in this paper), the continuous field approach (SPDE) is an efficient approach for both point‐reference data (e.g., continuous data that involves point samples from a continuous spatial distribution, such as birds’ observations in this paper) and areal data to model spatially smooth behavior (Lindgren, INLA discussion forum). See more about these two approaches in the section 5.1.

#### 3.2.3 Spatial models for data without excess zeros

For the data without excess zeros, we assumed a model with Gamma likelihood, defined by equations (2a) and (2b), where the linear predictor was defined by equation (1a) excluding the period effect.

#### 3.2.4 Joint modeling a covariate with misalignment

The predictive joint modeling with misalignment (Chapter 7 in Krainski et al., 2015) was applied to the seabird and sandeel observations, herring abundance, and density/usage maps with finer resolution grids (6*6 km, 5*5 km, and 1*1 km), where the species point locations did not match with the bio/physical variable locations.

Let $y=\left(y_{1},\ldots ,y_{n}\right)$ denote a response (observations, abundance, density, or usage) that is observed at $s_{y}=\left(s_{y1},\ldots ,s_{yn}\right)$ locations. Let $x=\left(x_{1},\ldots ,x_{m}\right)$ be a covariate (a bio/physical variable) at $s_{x}=\left(s_{x1},\ldots ,s_{xm}\right)$ locations. Let us also assume that ***x*** and ***y*** are having distributions in exponential family with means $\mu_{xj}=E\left(x_{j}|f\right)=h\left(f_{j}\right)$ and $\mu_{yi}=E\left(y_{i}|x,z,\beta \right)\left(i=1,\ldots ,n\;;\;j=1,\ldots ,m\right),$ respectively, where $\mu_{yi}$ is linked to the linear predictor φ_*i*_ via

$$\mu_{yi}=g\left(\varphi_{i}\right)$$

$$\varphi_{i}=\beta_{0}+\beta_{x}x_{i}^{*}+\vartheta_{i}$$

Here, $h\left(.\right)$ and $g\left(.\right)$ are monotonic inverse link functions, $f_{j}$ is a random field, β_0_is an intercept, β_*x*_ is a regression coefficient on covariate $x,x_{i}^{*}$ is the covariate at location of $y_{i}$, and *ϑ*_*i*_ is a zero mean random field.

The estimation process is done jointly for the ***x*** and ***y*** spatial models to predict bio/physical variables on the species locations. More information on the predictive joint modeling with misalignment and a detailed example of R code can be found in Krainski et al. (2015).

### 3.3 Model terms and priors

#### 3.3.1 Linear versus nonlinear effects of the covariates

We compared models with nonlinear effects of covariates with the models that have either only linear effects (when $f_{\lambda k_{1},i}\left(x_{k_{1}s,i}^{\prime }\right)=x_{k_{1}s,i}^{\prime }$ and $f_{pk_{2},i}\left(x_{k_{2}s,i}^{\prime }\right)=x_{k_{2}s,i}^{\prime }$) or a mixture of linear and nonlinear effects (see section 4).

#### 3.3.2 Prior choice

The choice of hyperparameters (parameters of prior distributions) for the spatially structured effect determines the smoothness of the spatial effect and spatial scale at which it operates and therefore these priors have to be chosen very carefully to avoid overfitting (Illian, Sørbye, & Rue, 2012). This is particularly crucial when working with spatial point patterns with relatively small number of points (Illian et al., 2012).

In this paper, we used large datasets (more than 12,000 data points each), except the herring data (across years), which had 171 and 345 data points for ages 1 and 2 & 3, respectively.

Here, we compared different priors to investigate whether there is a overfitting problem and how much the choice of priors helps to avoid overfitting.

By default, the BYM model in R‐INLA has minimally informative priors that are specified as log Gamma on the log of the unstructured effect precision and on the log of the structured effect precision.

We approached the problem of overfitting by choosing the priors so that the spatial effect operated at a similar spatial scale as selected covariates following Illian et al. (2012) and compared the model results based on these priors with the model results based on the default priors. Obtaining these priors was done by repeatedly fitting a model using different values for the shape parameter of the log Gamma prior and comparing the estimated spatial effect to a plot of the covariate (Illian, Sørbye, Rue, & Hendrichsen, 2010). For more information about how to approach the problem of overfitting and select priors so that the spatial effect operated at a similar scale as selected covariates, see Illian et al. (2010, 2012).

In addition, we also compared the joint‐species models’ results and herring single‐species models’ results based on the priors discussed above with the corresponding models with the priors based on the recent “penalized complexity prior” framework developed by Simpson, Rue, Riebler, Martins, and Sørbye (2017). In the “penalized complexity prior,” framework proper priors are defined to penalize the complexity induced by deviating from the simpler base model and are formulated after the input of a user‐defined scaling parameter for the model component (Simpson et al., 2017). For more information about the penalized complexity priors, see Simpson et al. (2017).

#### 3.3.3 Spatial confounding

Spatial confounding between the spatially structured effects (random effects) and fixed‐effect covariates showed that it can be strong enough that estimates of the fixed‐effect coefficients may change significantly when a spatially structured effect is included (Hodges & Reich, 2010). Hodges and Reich (2010) show how to avoid this spatial confounding by restricting the spatial random effect to the orthogonal complement of the fixed effects. We followed Hodges and Reich (2010) when linear effects of the covariates were considered.

### 3.4 Model selection

Due to the size of the datasets and limitations in computer power, we first examined the single‐species models to select the best habitat models for each species. We considered all possible combinations of covariates (bio/physical variables) excluding the combinations with highly (>0.6) correlated variables (e.g., BT was strongly correlated with NPP and PEA. NPP was also highly correlated with PEA). The goodness of fit for all the single‐species models with all the considered combinations of covariates was assessed using the deviance information criterion (DIC). The models with the lowest DIC values were considered as the best models.

We performed all computations using the R‐INLA package (Lindgren & Rue, 2015; Rue et al., 2009, 2014).

## 4 RESULTS

### 4.1 INLA and spatiotemporal zero‐inflated and hurdle models

Using INLA methods enabled us to fit these complex zero‐inflated and hurdle joint models at relatively little computational cost, while it could be computationally expensive to implement this with MCMC methods (Rue et al., 2009).

The zero‐inflated spatiotemporal models showed lower DIC values than the hurdle spatiotemporal models for the count temporal data with excess zeros. The zero‐inflated negative binomial models (ZINB) demonstrated lower DIC and fitted better than the zero‐inflated models with Poisson distribution (ZIP). We also compared the ZINB models with the negative binomial distribution (NB) models (results not shown). It was found out that ZINB models fitted better than NB models providing the lower DIC values.

The residual plots (not shown) for the single‐ and joint‐species’ models did not show any significant residual spatial structure indicating that the models probably explain all the spatial structure in the data.

#### 4.1.1 Prior choice

All the single‐species models, excluding two herring (across years) models for two age classes, showed the same best model selection results (Table 1), but different DIC values (not shown). Age 1 herring model (across years) that was based on the default priors showed that the DIC‐best model was the one with CHL and NPP as covariates, whereas the age 1 herring model (across years) based on the priors when the spatial effect operated at a similar scale as selected covariates or based on the penalized complexity prior framework, which included NPP, SP, and DVV (Table 1). Ages 2 and 3 herring model that was based on the default priors showed the same DIC‐best model results (Table 1) replacing the DVV variable with SP, whereas the one based on the penalized complexity priors included only CHL and NPP variables. Although the herring single‐species model selection results were slightly different for different prior choice, these results did not change the main conclusions (see sections 4.2, 5, and 6 below).

**Table 1:** DIC‐based single‐species model selection results

Species	Model	L	Covariates						DIC
			BT	CHL	NPP	PEA	SP	DVV	
Grey seals	BM^a^	G							−189,481.2
Harbour seals	H,BM^a^	G							−119,244.0
	H,BM^a^	B							
Porpoises, 1994	BM^a^	G							−146,944.0
Porpoises, 2005	BM^a^	G							−148,706.5
Herring (age1), across years	BM^a^	G							−1,179.0
Herring (age1)	H,BM^a^	G							604.07
	H,BM^a^	B							
Herring (ages 2 and 3), across years	BM^a^	G							−668.7
Herring (ages 2 and 3)	H,BM^a^	G							1,612.6
	H,BM^a^	B							
Sandeels, density	H,BM^a^	G							−9,9650.3
	H,BM^a^	B							
Sandeels, observations	H,SM	P							−7,5449.8
	H,SM	B							
Sandeels, observations	ZIP,SM	Z							−7,5491.1
Sandeels, observations	ZIP,SM	N							−75,536.2
Northern gannet, density	BM^a^	G							−180,365.5
Northern gannet, obs.	H,SM	P							−46,043.1
	H,SM	B							
Northern gannet, obs.	ZIP,SM	Z							−46,090.8
Northern gannet, obs.	ZIP,SM	N							−46,101.3
Common guillemot, density	H,BM^a^	G							−146,997.0
	H,BM^a^	B							
Common guillemot, obs.	H,SM	P							−57,765.4
	H,SM	B							
Common guillemot, obs.	ZIP,SM	Z							−57,801.2
Common guillemot, obs.	ZIP,SM	N							−57,848.1
Black‐legged kittiwake, density	BM^a^	G							−17,9252.4
Black‐legged kittiwake, obs.	H,SM	P							−43,405.5
	H,SM	B							
Black‐legged kittiwake, obs.	ZIP,SM	Z							−43,452.2
Black‐legged kittiwake, obs.	ZIP,SM	N							−43,481.8

Only the best‐supported models are shown and variables included in the best models are shaded in grey. Selected models for harbor porpoises are given separately for two different years (1994 and 2005) and for herring are given for different age groups (age 1 and ages 2 and 3). The biologic and physical variables are: bottom temperature (BT), maximum chlorophyll_a (CHL), net primary production (NPP), potential energy anomaly (PEA), depth‐averaged current speed (SP), and depth‐averaged vertical velocity from surface (DVV). L refers to likelihood model (B‐Binomial; G‐Gamma; P‐Poisson; Z‐zero‐inflated Poisson; N‐negative binomial). H refers to the hurdle models, ZIP refers to the zero‐inflated Poisson models. BM refers to the Besag–York–Mollie models for spatial effect, whereas SM refers to the stochastic partial differential equation models.

obs., observations.

a The SPDE and BYM models produced nearly identical results; as the datasets were identical we present only the BYM model selection results.

All the joint‐species models were not sensitive to the prior choice and produced nearly identical joint spatial trends (Figures 3, 4, 5) as well as other results (not shown) for all the types of priors (default priors, penalized complexity priors, and the priors when the spatial effect operated at a similar scale as the selected covariates).

### 4.2 Model selection

#### 4.2.1 Important single‐species habitat variables

**Figure 2:**
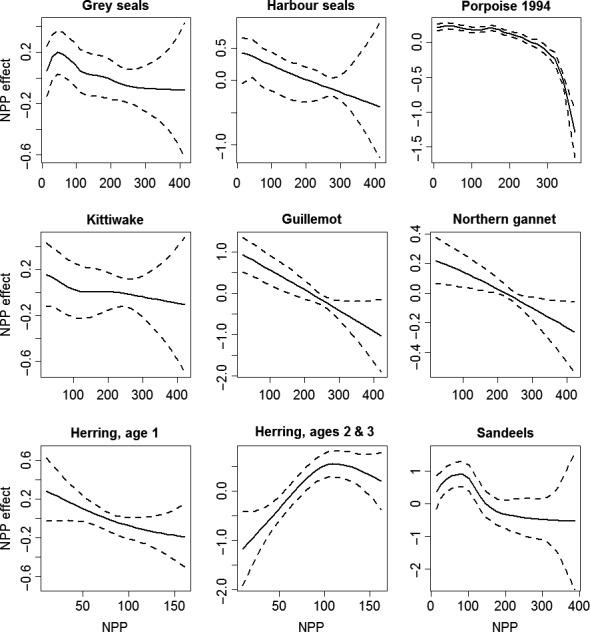
Estimated effect of (NPP) on grey seal usage (top left), harbor seal usage (top center), harbor porpoise density (1994 year) (top right), black‐legged kittiwake density (middle left), northern gannet density (middle center), common guillemot density (middle right), herring abundance, age1 (bottom left), herring abundance, ages 2 & 3 (bottom center) and sandeels (bottom right). The estimated effect is presented as smooth functions with 95% credible intervals (dashed lines) using BYM models

DIC‐based single‐species model selection results are found in Table 1. Only the best‐supported models are shown and they have DIC differences greater by at least 7 units than the next best model. The model selection results demonstrate that NPP (Figure 2) plays a vital role in determining habitat preferences of all the eight selected marine species. All other bio/physical variables showed importance for 2–5 species.

It should be noted here that DIC‐based joint‐species model selection results (Sadykova et al., 2017), where the competing or predator–prey species are assumed to share one set of bio/physical parameters, are producing somewhat different results from the DIC‐based single‐species model selection results. This might imply that single‐species habitat selection might differ from the joint‐species habitat selection (Sadykova et al., 2017).

For all single‐species models, the models with RW2 priors on NPP, DVV, and the effort variable gave the smallest DIC value, whereas the models with RW1 priors on PEA and the period variable gave the smallest DIC value. Most of the single‐species models gave smallest DIC value with RW2 priors on CHL, SP, and BT, but several models (CHL: grey and harbor seals; SP: sandeels, guillemot, and kittiwake; BT: grey seals, sandeels, and kittiwake) showed smallest DIC with RW1 priors on CHL, SP, and BT.

Bio/physical habitat preferences are found in Table 2. These preferences show bio/physical variable ranges with positive estimated effect on the species usage/densities/abundance from the DIC‐best BYM or SPDE models. The results show that, of the prey species, age 1 herring prefer NPP values from 9.8 to 89.3 mgC/m^2^, whereas ages 2 and 3 herring prefer the range from 72.5 to 154.2 mgC/m^2^, which indicates that herring of different ages prefer to live in two different habitats, which have a small overlap.

**Table 2:** Bio/physical habitat preferences from BYM models for spatial effect

Species	BT	CHL	NPP
Grey seal		(1.8, 21.3)	(14.7, 192.5)
Harbor seal	(9.4, 17.0)		(14.6, 201.2)
Harbor porpoise,1994			(8.9, 267.5)
Harbor porpoise, 2005	(6.6, 17.0)		
Herring, age1			(9.8, 89.3)
Herring, ages 2 and 3		(3.1, 19.3)	(72.5, 154.2)
Sandeels		(2.3, 24.2)^a^	(13.3, 177.6)
Northern gannet	(8.3, 15.9)	(1.80, 18.63)	(20.3, 214.4)^a^
Common guillemot			(20.5, 213.9)
Black‐legged kittiwake			(20.3, 214.5)

	PEA	SP	DVV
Harbor seal		(0.01, 0.22)	
Harbor porpoise, 1994			(−24.94, 23.27)
Herring, age1		(0.10, 0.26)	(−4.33, 5.99)
Herring, ages 2 and 3			(−4.32, 3.26)
Sandeels			(−21.69, 27.09)
Northern gannet		(0.05, 0.21)	
Common guillemot	(−0.01, 169.3)^a^	(0.01, 0.22)	(−17.79, 18.05)^a^
Black‐legged kittiwake	(−0.01, 162.2)^a^		(−27.61, 29.26)^a^

These preferences show bio/physical variable ranges with positive estimated effect on the species densities. Densities refer to the density maps (porpoise/sandeels/seabirds) or usage maps (seals) or abundance maps (herring).

a We also show here the variables that were not included in the best BYM models (density maps) but were included in the best SPDE models (observation data) to provide full habitat preferences.

Maximum and minimum values of the estimated nonlinear effect of the covariates (RW1 or RW2) on the selected species with 95% pointwise credible intervals are found in the Appendix S2. Estimated effect of NPP are found in Figure 2. The results demonstrate that NPP has the strongest estimated effect on sandeels, ages 2 and 3 herring, common guillemot, and harbor porpoise (in 1994) (Figure 2). The second most important variable was the DVV, where we can only rely on the estimates inside the [−1, 1] values as there were narrow 95% credible intervals of the mode/mean estimated effects within those value and a lack of the animal data outside that interval [−1, 1]. BT has the strongest estimated effect on harbor seals and harbor porpoise (2005); CHL on ages 2 and 3 herring. Some variables showed almost no or moderate effect (see Table S2 in Appendix S1).

#### 4.2.2 Single model selection: linear versus nonlinear effects of the covariates

The linear–nonlinear model comparison implemented for the single‐species BYM models showed that the best DIC model was often a model with a mixture of linear and nonlinear effects (7 out of 10 models), two DIC best single‐species models (porpoise 2005 and common guillemot) were the models with only linear effects of covariates and one DIC best single‐species model (black‐legged kittiwake) was a model with only nonlinear effects of covariates (Table S3 in Appendix S1).

This model selection results also demonstrate that NPP plays a vital role in determining habitat preferences (for six out of eight selected marine species)—either as linear or nonlinear covariate, which provides extra support to our previous findings. It also reveals that CHL plays a significant role (for six out of eight species), mainly as a fixed effect.

In this paper, only results from the models with nonlinear effects (Table 1) are shown as our research interest focuses on inference about those nonlinear smooth functions and detection of the bio/physical species habitat preferences that can only be obtained using nonlinear effects.

#### 4.2.3 Joint model outcomes: common spatial trends

**Figure 3:**
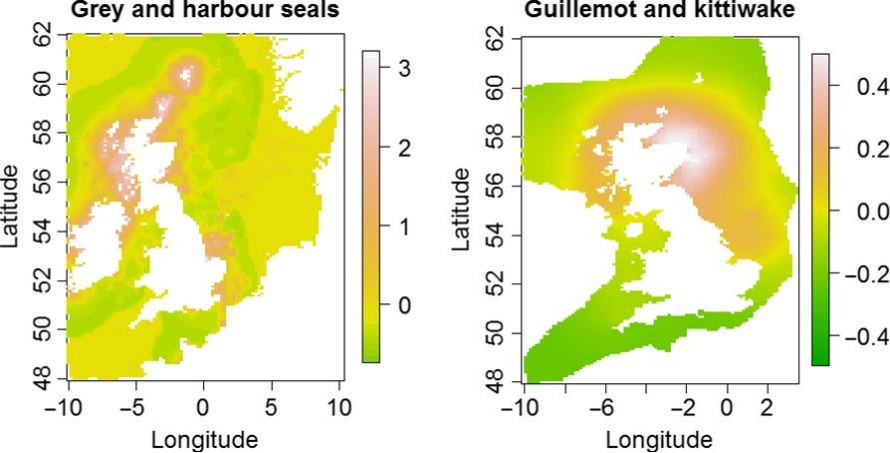
Estimated common spatial trends (posterior mean) for competing species: (1) grey seals and harbor seals (left) and (2) common guillemot and black‐legged kittiwake (right)

**Figure 4:**
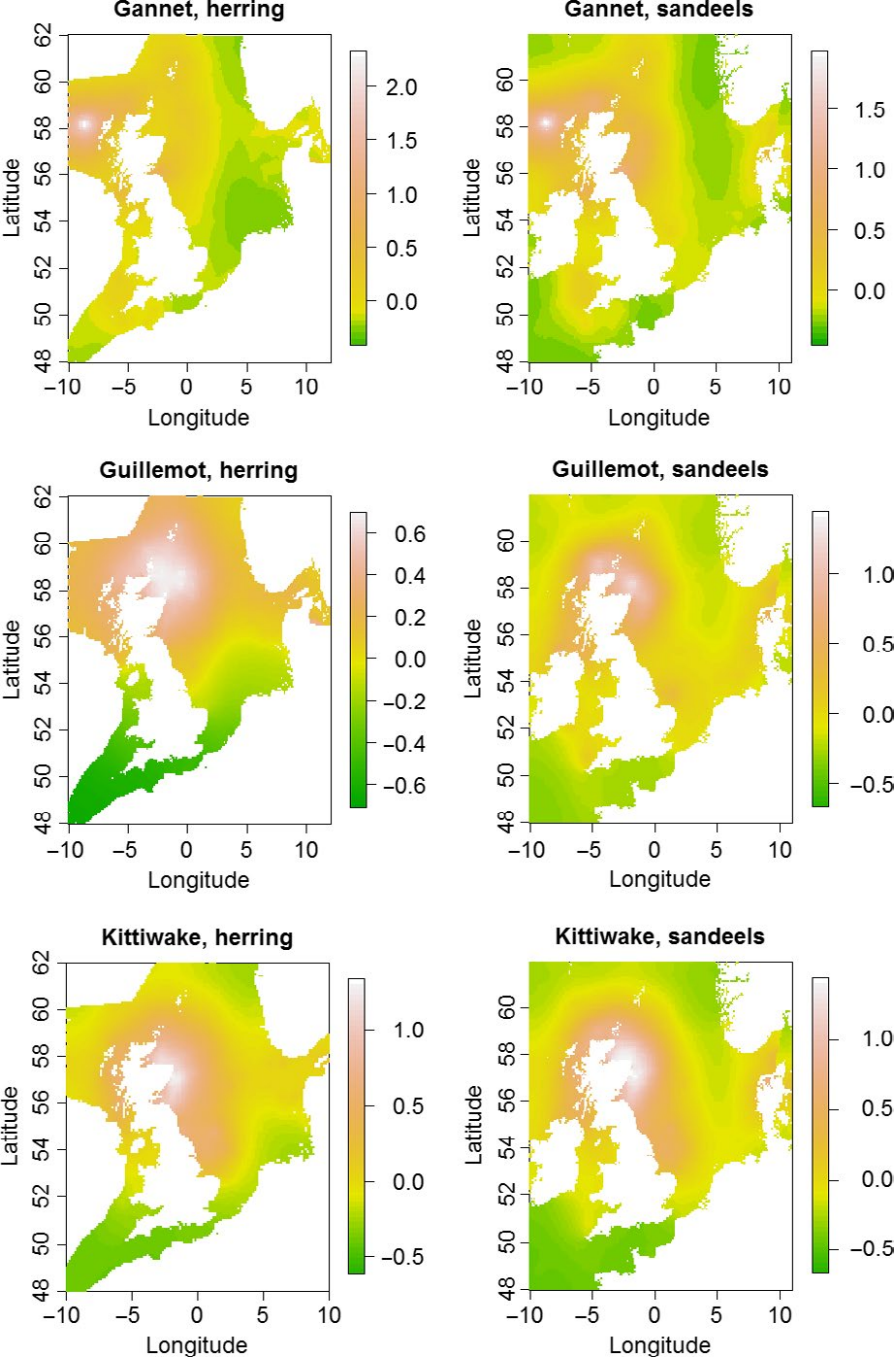
Estimated common spatial trends (posterior mean) for predator–prey species: (1) northern gannet and herring (all ages) (top left), (2) northern gannet and sandeels (top right), (3) common guillemot and herring (all ages) (middle left), (4) common guillemot and sandeels (middle right), (5) black‐legged kittiwake and herring (all ages) (bottom left), and (6) black‐legged kittiwake and sandeels (bottom right)

**Figure 5:**
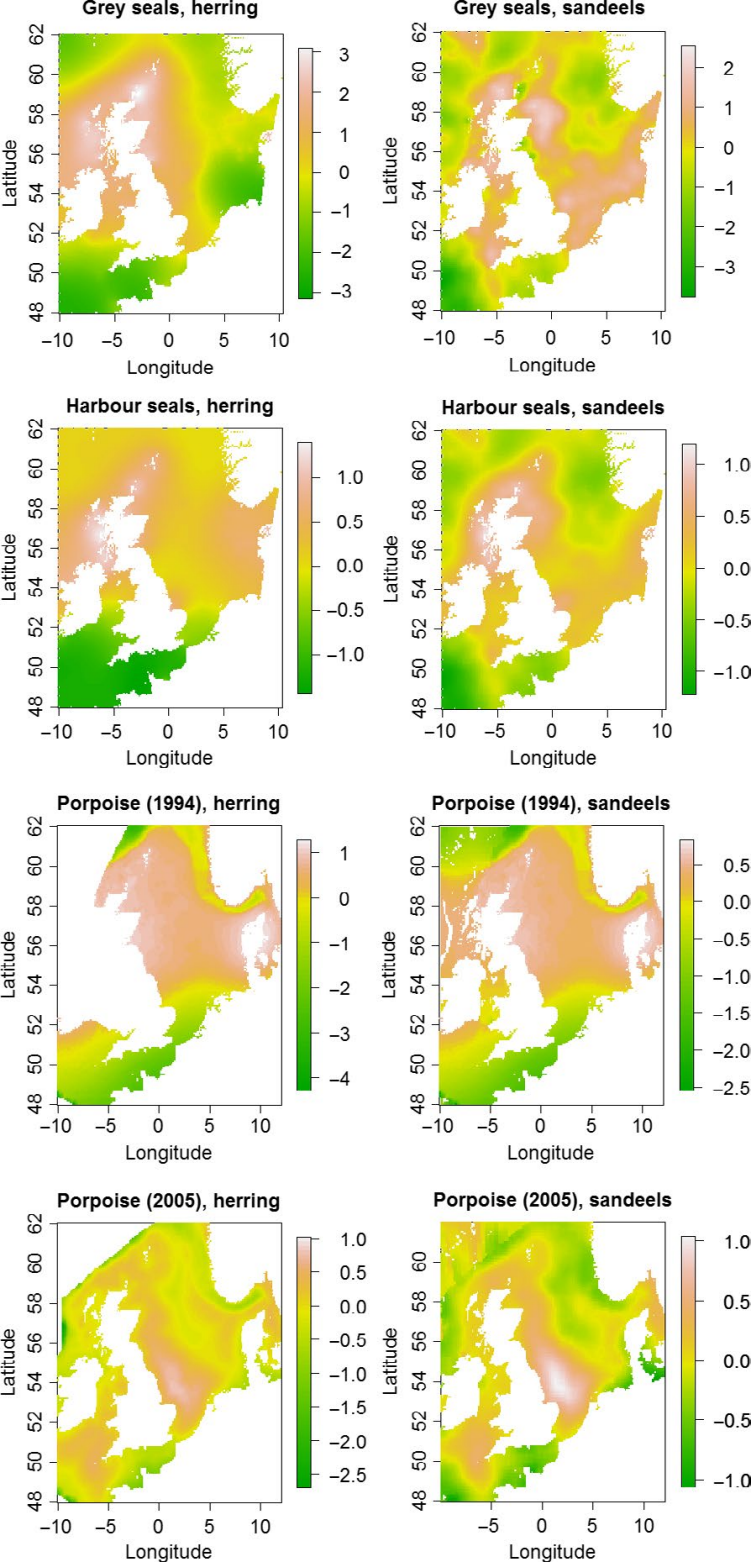
Estimated common spatial trends (posterior mean) for predator–prey species: (1) grey seals and herring (all ages) (top left), (2) grey seals and sandeels (top right), (3) harbor seals and herring (all ages) (middle top left), (4) harbor seals and sandeels (middle top right), (5) porpoise (1994 year) and herring (all ages) (middle bottom left), (6) porpoise (1994 year) and sandeels (middle bottom right), (7) porpoise (2005 year) and herring (all ages) (bottom left), and (8) porpoise (2005 year) and sandeels (bottom right)

The estimated common spatial trends, in other words, residual spatial autocorrelation unexplained by covariates, for competing and predator–prey species are seen in Figures 3, 4, 5. The white and pink areas (with values >0) of these smooth common spatial trends identify the high activity areas of the coupled species. In these joint models, herring of different ages were regarded as separate species such that the joint models with herring and predator species essentially contained three species.

In all, 14 of the 16 pairwise joint models showed a range from 2 to 6 on the spatial effect (Figures 3, 4, 5) (where “range” is the difference between the highest and lowest spatial effect values). Considering also that the standard deviations of the random fields (figures not shown) were from 0.2 to 0.7 for these joint modes, the spatial dependence is significant (Krainski et al., 2015). Those species combinations that show particular high co‐spatial dependence are grey and harbors seals, grey seals and both herring and sandeels, gannet and both herring and sandeels. Nine pairs of predators and prey show moderate spatial dependence and the pairs that do not show significant spatial dependence are guillemots and kittiwakes and guillemots and herring. Here, we would like to mention that the last result might also be due to the fact that some of the bio/physical variables explain some of the spatial structure in these data pairs.

The spatial effect look smooth, without showing any local structure in the spatial effect (i.e., without showing clustering at a smaller scale than the selected covariates) (Figures 3, 4, 5), which might suggest that the risk of overfitting is low.

The intercept posterior means and SD are not shown due to a large number of considered models.

## 5 DISCUSSION

### 5.1 Methodological discussion

In this paper, we showed that INLA is an efficient approach to fit complex joint spatial models and successfully created complex spatial models with several types of data from different sources and differing qualities. The hurdle and zero‐inflated models provided useful frameworks for modeling data with excess zeros. The constructed models were able to identify, from the selection of important bio/physical habitat variables that will change with climate change and large‐scale anthropomorphic activity, common spatial trends for a range of important competing and prey–predator species.

Using INLA methodology has a number of advantages, such as low computational cost, where MCMC algorithms need hours and days to run, INLA approximations provide more precise estimates in seconds and minutes (Rue et al., 2009). Another advantage of the INLA approach is its possibility to perform complex Bayesian spatial models in an automatic, streamlined way, to compute model comparison criteria and various predictive measures so that different models can be compared (Rue et al., 2009). In addition, INLA may be used to fit a large class of latent Gaussian models in a Bayesian framework (Rue et al., 2009). But there are few drawbacks in using INLA that should be noted. First, the computational cost is exponential with respect to the number of hyperparameters (Blangiardo & Cameletti, 2015). A second issue is that although the R‐INLA package is updated regularly, not every model type is currently available through the R‐INLA interface (Martins, Simpson, Lindgren, & Rue, 2013).

Although the zero‐inflated and hurdle models are very useful, it should be noted that they also have some important limitations. First, the zero‐inflated and hurdle models are often over‐parametrized due to the complex nature of the parametrization. When the number of parameters is nearly doubled, it might be more difficult to interpret them. Finally, both hurdle and zero‐inflated models are based on assumptions regarding the process of how zero observations are generated and these assumptions are difficult to validate.

As we also mentioned in the “hurdle spatial and spatiotemporal models” section, using SPDE is an efficient approach for both point‐reference data and confounding data to model spatially smooth behavior (Lindgren, INLA discussion forum), whereas the BYM approach is common for the areal datasets as it is quite difficult to construct a conditional autoregressive model on an irregular lattice that is resolution‐consistent (Rue & Held, 2005; Simpson, Illian, Lindgren, Sørbye, & Rue, 2016). In this paper, we found that the BYM approach is slightly more computationally convenient for the areal data. However, as Lindgren (2013) writes about the SPDE approach “when building and using hierarchical models with latent random fields it is important to remember that the latent fields often represent real‐world phenomena that exist independently of whether they are observed in a given location or not. Thus, we are not building models solely for discretely observed data, but for approximations of entire processes defined on continuous domains.” Thus, the SPDE approach might be preferred when one is interested in modeling the entire domain of interest or when there are several disconnected components in the map so that the model is well defined even when there are missing data (Lindgren, INLA discussion forum).

#### 5.1.1 Linear versus nonlinear effects of the covariates

In this paper, we selected models with nonlinear effects due to our research focus on the detection of the bio/physical species habitat preferences and inference about those nonlinear relationships. Additionally, using nonlinear effects is important in order to evaluate how species habitat preferences are going to transform with modifications in bio/physical variables due to climate change and large‐scale anthropomorphic activity.

However, it should be noted that using linear effects or treating some of the effects as linear is reducing computational time significantly and avoids overfitting problem that might arise when using nonlinear effects.

#### 5.1.2 Prior choice

This paper confirmed that the prior choice might be vital especially when dealing with relatively small number of points (Illian et al., 2012).

The authors strongly recommend to use either the penalized complex prior framework as it might give improved control on the influence of the prior choices compared with traditional priors (Simpson et al., 2017) or choosing the priors so that the spatial effect operated at a similar spatial scale as selected covariates (Illian et al., 2012).

### 5.2 Ecological implications

#### 5.2.1 Single habitat variables

Important ecological outcomes of this analysis reveal that a biological variable, NPP, plays a most significant role in determining habitat preferences of all the selected marine species. Interestingly, NPP has the strongest effect on the selected prey species (sandeels and herring), showing optimal or positive relationships (with 2‐ & 3‐year old herring), but showed mostly a negative relationship with all the predator species. This result suggests that the prey and predators are selecting aspects of this habitat type very differently and that might be a reflection of prey species avoiding areas with predators hence appearing as a repulsive effect of predator on prey. Therefore, future climate (Holt, Butenschon et al., 2012; Holt, Hughes et al., 2012) or anthropogenic forces (De Dominicis et al., 2017; Van der Molen et al., 2016; Wakelin et al., 2015) acting on this shared important habitat variable could have an important effect on the range of overlap of predator and prey species.

The second most common variable, shared across seven species, was a physical variable, DVV, which indicates there is an association with vertical speeds in the water column and may be due to the presence of shear between water layers which may provide a role in prey capture (Scott, Webb, Palmer, Embling, & Sharples, 2013). Both CHL and SP were important to four species, and the other remaining two physical variables, BT and PEA were important to three and two species, respectively. The importance of biologic parameters over that of physical ones may suggest that biologic parameters are more reliable habitat variables as they are essentially integrators of an additive range of single physical conditions. Therefore, while the biologic parameters are not necessarily accurate predictors in absolute value, they seem to be the better predictor variables for mobile species than individual physical parameters.

#### 5.2.2 Joint model predictions of common spatial trends

Identifying the locations of common spatial trends for competing and predator–prey species allows ecologists and managers to quantify the degree of spatial overlap for these pairs of species and can provide a more comprehensive basis for understanding common spatial habitats. This knowledge will allow more accurate predictions of the separate effects of climate change and other anthropogenic effects that are large enough to alter marine habitats such as the large‐scale extraction of tidal, wave, and wind energy. Of the competing sets of species, we expected to have common areas of usage, the two seals species, grey and common, showed significant spatial dependence. However, the two bird species, guillemot and kittiwakes, did not which may be due to the fact that some of the bio/physical variables explained some of the spatial structure for the pair, but it also might be that despite them both foraging for similar prey species, their foraging techniques are so different (unlike the pair of competing seal species) that they forage primarily in different spatial regions and therefore do not have a strong significant spatial dependence. If the last case is true, then this is intriguing as the seal species had less bio/physical variables in common (only NPP) than the bird species did (NPP, PEA, and DVV). Therefore, this result indicates that just using information on the range of shared important individual physical variables is not enough information to estimate which species will have common spatial usage and that joint models provide valuable non‐intuitive insights.

For the common trends in predator–prey combinations, there were significant spatial dependences for most of the other 14 pairs (Figures 4 and 5). The stronger spatial dependences between predator–prey species pairs were those that shared significant relationships with both biological variables (CHL and NPP). Those relationships were between gannets and herring as well as sandeels, and grey seals and herring. This is the case although the individual relationships between the different species and biologic variables were quite different (Figure 2). The weaker, but still significant spatial dependences for the other seven predator–prey species pairs had only NPP or DVV in common. Those relationships were between the predators kittiwakes and porpoise and both prey species of herring and sandeels, as well as harbors seals and only sandeels. The indications of these results are that the range of predator–prey species pairs have different important habitat variables making up their common spatial trends. Therefore, the differing effects of both climate change and energy extraction may have very complex effects on where they will overlap in the future.

## 6 CONCLUSION

In summary, we recommend the approach of using INLA with zero‐inflated and hurdle models in the exploration of identifying important bio‐physical variables in joint spatial usage between marine mobile competing and predator–prey species. This type of approach is relevant for numerous issues in the management and conservation of mobile marine species, and is a comprehensive basis for understanding common spatial habitats. Studying multispecies spatial interactions might bring extra knowledge about consequences for the dynamics of marine species in a bio/physical environment that is changing rapidly. Joint models, considered in this paper, can be used for different purposes of interest to ecologist such as providing predictions of species distributions, making inferences about environmental effects or environment–species interaction. In addition, an integrated analysis (joint modelling) is often used to increase precision of parameter estimates as information may be “borrowed” across different datasets (Illian et al., 2013). These joint models are becoming increasingly common (Illian et al., 2013; King, Morgan, Gimenez, & Brooks, 2009).

The biologic and physical variables used in this study are those that will change with predicted climate change and large‐scale energy extraction. By demonstrating how to calculate current competing and predator–prey joint distributions, the proposed approach will be especially useful for separating out the change in the level of predicted overlap in species distributions in the future due to either or both of climate change and energy extraction. What is important to do next is to evaluate the extent of change in each of these bio/physical variables under different future scenarios and assess the subsequent joint spatial overlaps to evaluate if there is a contrasting or synergistic interplay between climate change and energy extraction that is better or worse for ranges of competing and predator–prey species.

## CONFLICT OF INTEREST

None declared.
