# Supplementary material for: Bayesian joint models with INLA exploring marine mobile predator–prey and competitor species habitat overlap
Source: Ecol Evol. 2017 Jun 7;7(14):5212–26. doi: 10.1002/ece3.3081 (PMC5528225; doi:10.1002/ece3.3081)
Supplement: Supplementary file 1 [file ECE3-7-5212-s001.docx]

**Bayesian joint models with INLA exploring marine mobile predator-prey and competitor species habitat overlap – Appendix**

**D. Sadykova, B.E. Scott, M. De Dominicis, S.L. Wakelin, A. Sadykov and J. Wolf**

**November 20, 2016**

**Results**

**ADDITIONAL EFFECTS DUE TO UNEVEN AMOUNT OF EFFORT IN OBSERVATIONAL DATA**

**Figure S7 demonstrates the estimated effect of effort within the SPDE models on the species observations (black-legged kittiwake, northern gannet, common guillemot and sandeels) and the species abundance (all ages herring). The results indicate that effort has a positive effect on all the selected species and therefore the number of observations (or abundance) is going to be higher when more effort is concentrated in that area and needed to be taken into account.**

Table S1. Percentage of zeros in the data sets.

|  |  | **% of zeros** | |
| --- | --- | --- | --- |
| **Species** | **Type** | **Original** | **Reduced** |
| Grey seal | usage map | 0.3 |  |
| Harbour seal | usage map | 39.0 |  |
| Harbour porpoise, 1994 | density map | 0.0 |  |
| Harbour porpoise, 2005 | density map | 0.0 |  |
| Herring, age 1 | abundance map | 42.6 |  |
| Herring, age 1, across years | abundance map | 1.8 |  |
| Herring, ages 2&3 | abundance map | 37.6 |  |
| Herring, ages 2&3, across years | abundance map | 1.2 |  |
| Sandeels | density map | 23.0 |  |
| Sandeels | observations | 95.6 | 74.8 |
| Northern gannet | density map | 0.3 |  |
| Northern gannet | observations | 76.1 | 62.0 |
| Common guillemot | density map | 19.5 |  |
| Common guillemot | observations | 32.8 | 30.6 |
| Black-legged kittiwake | density map | 4.1 |  |
| Black-legged kittiwake | observations | 78.6 | 50.4 |

Table S2. Maximum and minimum values of the estimated non-linear effect of the covariates on grey and harbour seal usage^*^, porpoise densities (1994 and 2005 years), sandeels density^**^, herring (age 1 and ages 2&3)^***^, northern gannet^****^, common guillemot^****^ and black-legged kittiwake^****^ with 95% pointwise credible intervals. Max refers to the maximum estimated effect, min refers to the minimum estimated effect.

| **Species** | **Covariates** | **Max** | **Max CI** | **Min** | **Min CI** |
| --- | --- | --- | --- | --- | --- |
| Grey seal^*^ | CHL | 0.31 | (0.21, 0.40) | -0.28 | (-0.41, -0.04) |
|  | NPP | 0.20 | (0.03, 0.37) | -0.10 | (-0.62, 0.43) |
| Harbour seal^*^ | BT | 0.77 | (0.61, 0.93) | -0.65 | (-0.92, -0.38) |
|  | NPP | 0.44 | (-0.05, 0.66) | -0.45 | (-1.2, 0.89) |
|  | SP | 0.10 | (-0.05, 0.26) | -0.10 | (-0.29, 0.08) |
| Harbour porpoise, 1994 | NPP | 0.24 | (0.19, 0.28) | -1.29 | (-1.66, -0.97) |
|  | DVV | 0.02 | (0.01, 0.03) | -0.02 | (-0.06, 0.01) |
| Harbour porpoise, 2005 | BT | 0.63 | (0.59, 0.67) | -1.64 | (-1.78, -1.50) |
| Sandeels^**^ | CHL | 0.01 | (-0.02, 0.03) | -0.01 | (-0.02, 0.02) |
|  | NPP | 0.89 | (0.51, 1.28) | -0.53 | (-2.61, 1.55) |
|  | DVV | 0.02 | (-0.06, 0.10) | -0.02 | (-0.24, 0.20) |
| Herring, age 1 ^***^ | NPP | 0.12 | (-0.49, 0.72) | -0.14 | (-0.75, 0.48) |
|  | SP | 0.28 | (-0.96, 1.52) | -0.17 | (-0.92, 0.57) |
|  | DVV | 0.01 | (-0.95, 0.97) | -0.03 | (-1.62, 1.57) |
| Herring, ages 2&3 ^***^ | CHL | 0.94 | (0.30, 1.55) | -1.95 | (-3.22, -0.62) |
|  | NPP | 0.72 | (0.38, 1.05) | -1.15 | (-2.24, -0.06) |
|  | DVV | 0.01 | (-0.16, 0.20) | -0.03 | (-1.07, 1.03) |
| Northern gannet ^****^ | BT | 0.21 | (0.09, 0.31) | -0.16 | (-0.41, 0.12) |
|  | CHL | 0.03 | (-0.10, 0.15) | -0.05 | (-0.29, 0.16) |
|  | NPP | 0.22 | (0.07, 0.38) | -0.25 | (-0.53, -0.06) |
|  | SP | 0.03 | (-0.29, 0.21) | -0.02 | (-0.19, 0.31) |
| Common guillemot ^****^ | NPP | 0.94 | (0.52, 1.36) | -1.02 | (-1.9, -0.15) |
|  | PEA | 0.06 | (-0.19, 0.31) | -0.02 | (-0.97, 0.94) |
|  | SP | 0.05 | (0.03, 0.07) | -0.03 | (-0.12, 0.08) |
|  | DVV | 0.29 | (-0.07, 0.66) | -0.42 | (-0.95, 0.10) |
| Black-legged kittiwake^****^ | NPP | 0.19 | (-0.05, 0.42) | -0.19 | (-0.46, 0.09) |
|  | PEA | 0.02 | (0.01, 0.03) | -0.01 | (-0.02, 0.01) |
|  | DVV | 0.12 | (-0.68, 0.91) | -0.24 | (-1.30, 0.81) |

*Seal usage maps that were used in the construction of the models represent estimates of mean density of **hundred** seals per 7x7 km grid cells.

**Sandeels density represents **hundreds** of sandeels per 7x7 km grid cells.

***Herring abundance maps were used as **100 millions** of herring per 7x7 km grid cells.

****Seabirds densities were used as **hundreds** of birds per 7x7 km grid cells.

Table S3. DIC-based single-species Besag-York-Mollie (BYM) model selection results (comparing models with nonlinear, linear and a mixture of linear and nonlinear effects of covariates). Only the best supported models are shown and variables included in the best models are shaded in grey. Selected models for harbour porpoises are given separately for two different years (1994 and 2005) and for herring are given for different age groups (age 1 and ages 2&3). The biological and physical variables BT, CHL, NPP, PEA, SP and DVV. L refers to likelihood model (B-Binomial; G-Gamma). H refers to the hurdle models. Li refers to linear effects and N refers to nonlinear effects of covariates.

| Species | Model | L | Covariates | | | | | | DIC |
| --- | --- | --- | --- | --- | --- | --- | --- | --- | --- |
|  |  |  | BT | CHL | NPP | PEA | SP | DVV |  |
| Grey seals |  | G |  | Li | N |  |  |  | -189592.9 |
| Harbour seals | H | G | N | Li |  |  |  |  | -119420.3 |
|  | H | B |  |  | Li |  |  |  |  |
| Porpoises,1994 |  | G |  |  |  | Li |  | N | -146954.2 |
| Porpoises,2005 |  | G |  |  |  | Li |  |  | -148919.9 |
| Herring (age1), across years |  | G |  |  | Li |  |  | N | -1224.3 |
| Herring (ages2&3), across years |  | G |  | Li | N |  |  | Li | -698.6 |
| Sandeels, density | H | G |  | Li | N |  |  |  | -100002.6 |
|  | H | B |  |  |  | Li |  |  |  |
| Northern gannet, density |  | G | Li | N |  |  |  |  | -180404.3 |
| Common guillemot, density | H | G |  |  | Li |  |  |  | -147240.3 |
|  | H | B |  | Li |  |  | Li |  |  |
| Black-legged kittiwake, density |  | G |  |  | N |  |  |  | -179252.4 |


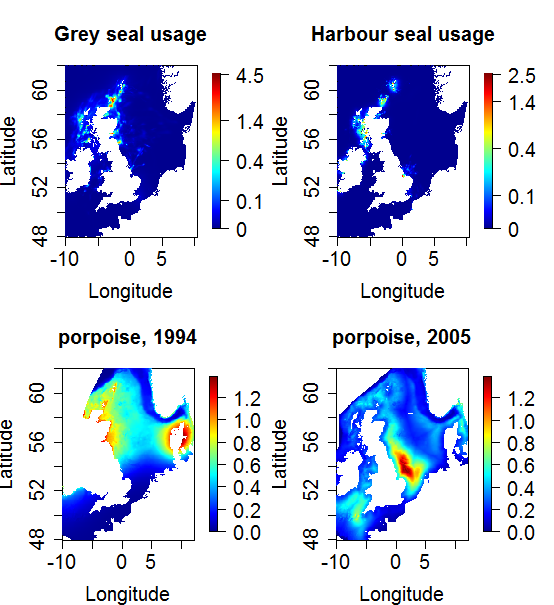


Figure S1. Grey seal usage map (top left), harbour seal usage map (top right), both are all year usage. Harbour porpoise density map (1994 year) (bottom left) and harbour porpoise density map (2005 year) (bottom right) from SCANS surveys in July of each year. The seal usage maps are given in hundreds of seals per 7*7 km grid cells. The porpoise density maps show porpoise density in individuals per 7*7 km.


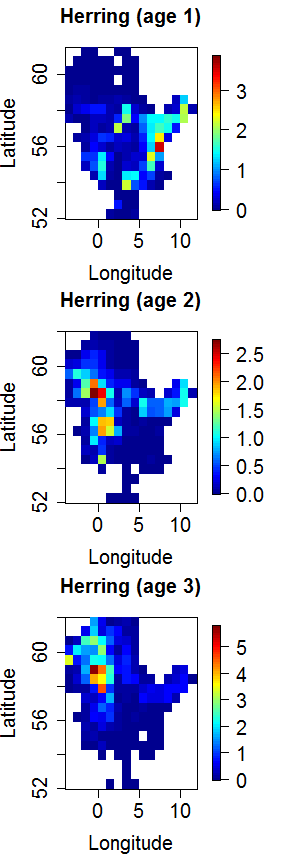


Figure S2. Herring abundance maps from acoustic surveys over 9 years (2003-2009, 2013, 2014 in 100 millions of herring per 56*56km grid cell) across 9 years: age 1 (top); age 2 (middle) and age 3 (bottom). All data come from month of July


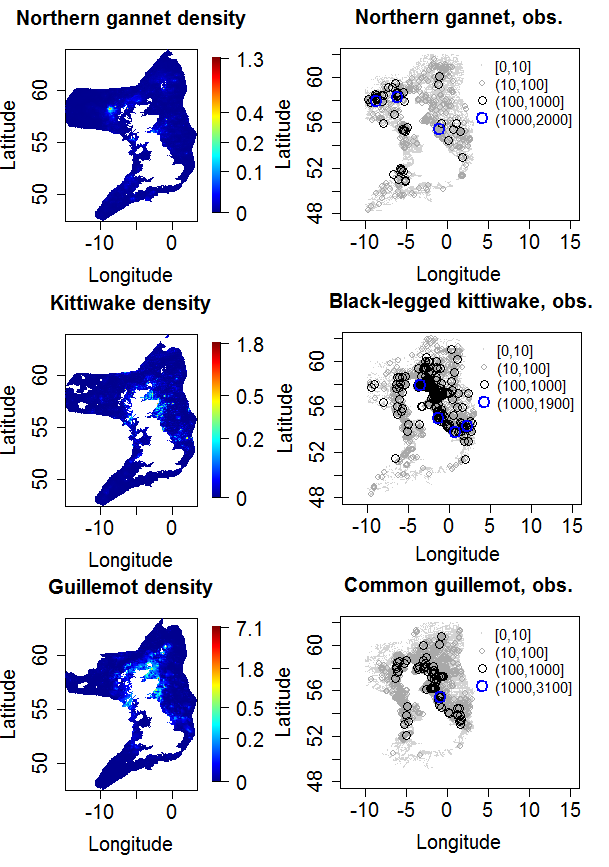


Figure S3. Northern gannet density map (top left), northern gannet count observations (top right), black-legged kittiwake density map (middle left), black-legged kittiwake count observations (middle right), common guillemot density map (bottom left), common guillemot count observations (bottom right). All the density maps are from 28 years (across all the seasons) and are given in hundreds of birds per 7*7 km grid cells.


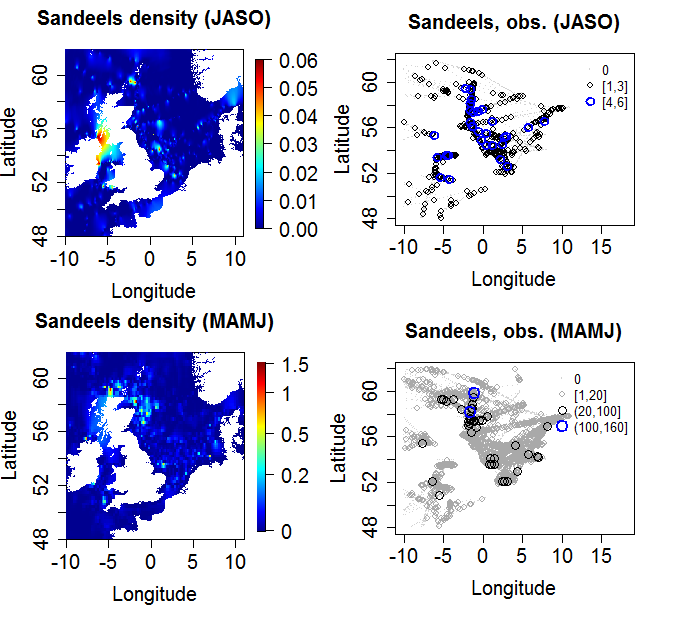


Figure S4. Predicted sandeel density (in hundreds of sandeels per 7*7 grid) surface maps from Poisson kriging (left) and sandeel count observations (right) during the spring season (March - June) (bottom) and during the summer season (July - October) (top) from CPR surveys.


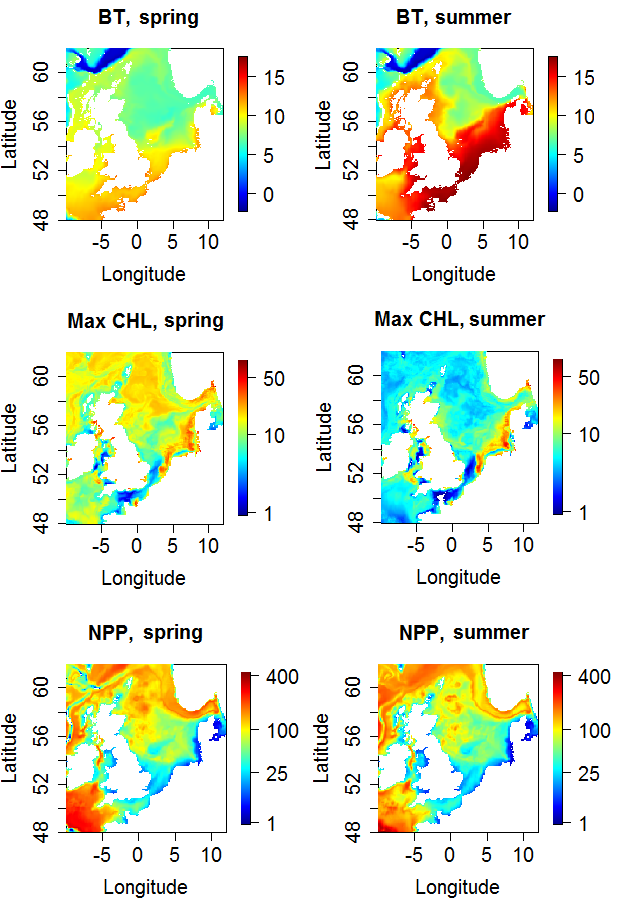


Figure S5. Bio/physical habitat variables: (1) bottom temperature (BT) (⁰C) spring season (top left), summer season (top right); (2) maximum chlorophyll a (CHL) (mg C/m^3^) spring season (middle left), summer season (middle right); (3) net primary production (NPP) (mgC/m^2^/day) spring season (bottom left), summer season (bottom right). Spring season represents March, April, May, June and summer season represents July, August, September and October.


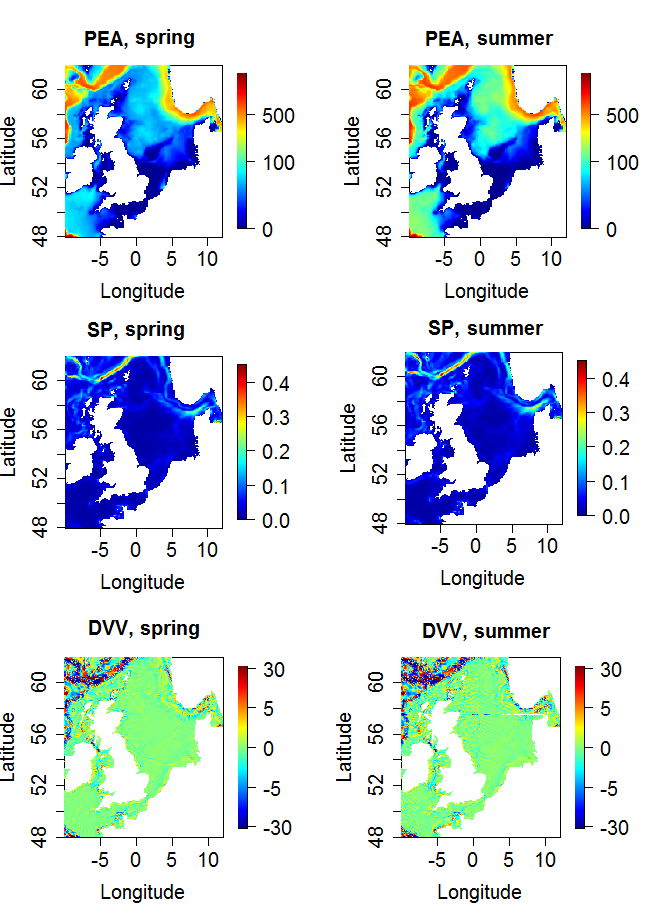


Figure S6. Bio/physical habitat variables: (4) potential energy anomaly (PEA) (J/m^3^) spring season (top left), summer season (top right); (5) depth-averaged current speed (SP) (m/s) spring season (middle left), summer season (middle right); (6) depth-averaged vertical velocity from surface (DVV) (m/day) spring season (bottom left), summer season (bottom right). Spring season represents March, April, May, June and summer season represents July, August, September and October.


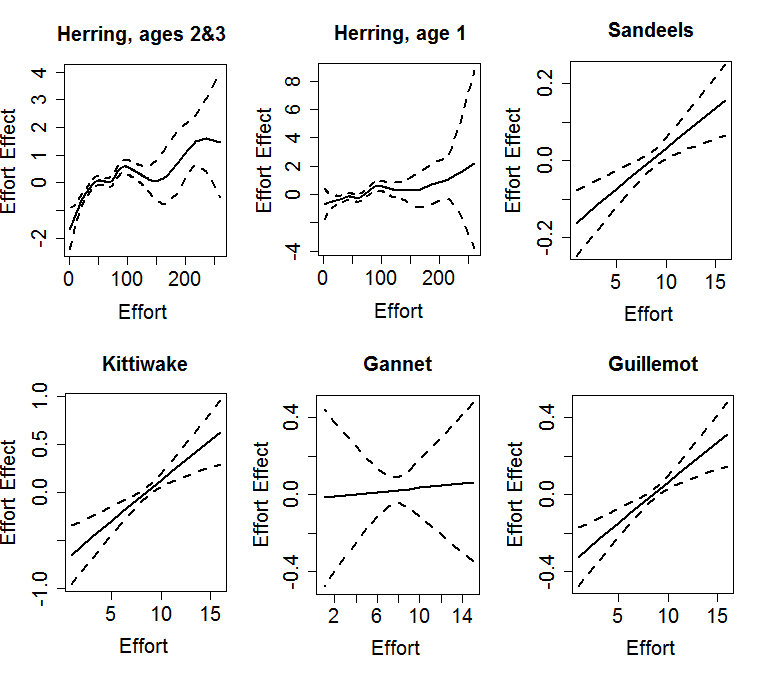


**Figure S7. Estimated effect of effort (amount of sampling) on herring abundance, ages 2&3 (top left), herring abundance, age 1 (top middle), sandeels observations (top right), black-legged kittiwake observations (bottom left), northern gannet observations (bottom middle) and common guillemot observations (bottom right).**
